# Electrochemical L-Tyrosine Sensor Based on a Glassy Carbon Electrode Modified with Exfoliated Graphene

**DOI:** 10.3390/s22103606

**Published:** 2022-05-10

**Authors:** Codruţa Varodi, Florina Pogăcean, Maria Coroş, Alexandra Ciorîță, Stela Pruneanu

**Affiliations:** National Institute for Research and Development of Isotopic and Molecular Technologies, Donat Street, No. 67-103, 400293 Cluj-Napoca, Romania; codruta.varodi@itim-cj.ro (C.V.); maria.coros@itim-cj.ro (M.C.); alexandra.ciorita@itim-cj.ro (A.C.)

**Keywords:** L-tyrosine, electrochemical exfoliation, graphene-modified electrode

## Abstract

In this study, a graphene sample (EGr) was synthesized by electrochemical exfoliation of graphite rods in electrolyte solution containing 0.1 M ammonia and 0.1 M ammonium thiocyanate. The morphology of the powder deposited onto a solid substrate was investigated by the scanning electron microscopy (SEM) technique. The SEM micrographs evidenced large and smooth areas corresponding to the basal plane of graphene as well as white lines (edges) where graphene layers fold-up. The high porosity of the material brings a major advantage, such as the increase of the active area of the modified electrode (EGr/GC) in comparison with that of bare glassy carbon (GC). The graphene modified electrode was successfully tested for L-tyrosine detection and the results were compared with those of bare GC. For EGr/GC, the oxidation peak of L-tyrosine had high intensity (1.69 × 10^−5^ A) and appeared at lower potential (+0.64 V) comparing with that of bare GC (+0.84 V). In addition, the graphene-modified electrode had a considerably larger sensitivity (0.0124 A/M) and lower detection limit (1.81 × 10^−6^ M), proving the advantages of employing graphene in electrochemical sensing.

## 1. Introduction

Tyrosine (Tyr) is a small, electroactive, and aromatic amino acid used to regulate the signal transduction process in proteins [1]. In humans, tyrosine is an important precursor of thyroid hormones, dopamine, adrenaline, and is used to establish and maintain a balanced nutrition [2,3]. It has been reported that a variation in the concentration of tyrosine can lead to severe chronic diseases such as tyrosinemia [4], and Parkinson’s disease [5]. Therefore, the detection of Tyr is very important for health assessment.

Several methods have been developed for the detection of L-tyrosine including surface-enhanced Raman scattering [6], high-performance liquid chromatography [7], and luminescence [8] methods. Even though these methods could achieve good determination results, many disadvantages have appeared in practical applications. For example, SERS measurement requires complex sample-making and sampling processes. HPLC involves high-cost columns and wastes of organic solvents. Luminescence detection is generally time-consuming and requires annealing and recalibration after each use, for accuracy. Also, most of these analytical methods need some special training for their operation, and the instruments for these procedures are relatively expensive. In this respect, electrochemical methods are good alternatives due to their sensitivity, accuracy, simplicity, and lower cost. A survey of the literature points out that there are some electrochemical methods developed for determining L-tyrosine. The larger part of these electrochemical methods involves the application of modified electrodes. Some examples of materials used to modify the electrodes includes copper oxide nanoparticles [9], gold nanoparticles [10], polymers [11], and tyrosinase multi-walled carbon nanotube polysulfone [12]. Recently, graphene-based materials have been widely used for electrode modification [13] due to their remarkable electro-catalytic properties.

Graphene, consisting of a monolayer of carbon atoms exhibits excellent mechanical, thermal, electrical, and optical properties [14,15]. Its large surface area may promote the adsorption of the target analyte, which is useful in various applications [16,17]. In electrochemistry, graphene and graphene-based materials deposited on top of a conductive substrate may substantially decrease the oxidation potential and thus enhance the electrochemical performance [18]. The direct exfoliation of graphite (rods or powder) in solution, such as electrochemical [19,20], sonochemical [21,22], or liquid-phase exfoliation [23,24] enable the fabrication of high-quality graphene via a low-temperature process. By employing different methods, the properties of graphene sample can be adjusted including the defect density, number of layers, and yield [25]. The electrochemical exfoliation of graphite rods is one of the most efficient methods for bulk production of graphene. The applied voltage transports the ionic species in the electrolyte to intercalate into the graphite layers and increase the inter-layer distance. According to the power supply applied on graphite electrodes, there are cathodic (employing a negative bias) and anodic (working with a positive bias) exfoliation methods [20]. Anodic exfoliation is the most common electrochemical method as a result of its high exfoliation efficiency which produces graphene in a relatively small period of time. Graphene based electrodes have been used for detection of different kinds of molecules [26]. For example, Wei et al. [27] used a graphene/Pt nanoparticles NPs modified glassy carbon electrode as a sensitive and simple sensor for simultaneous measurement of tryptophan and tyrosine in the presence of 5 hydroxytryptophan. A cost-effective reduced graphene oxide–copper hybrid nano thin-film modified pencil graphite electrode with excellent performance, good stability, and reproducibility has been employed to detect the L-tyrosine enantiomer [28]. Another electrochemical sensor developed for determination of tyrosine was based on graphene and gold nanoparticles modified glassy carbon electrode [29].

In this work, a simple approach was used for manufacturing a modified glassy carbon electrode employing electrochemically exfoliated graphene. The electrochemical properties of the modified electrode were investigated at electro-catalytic oxidation of Tyr. The electrochemically exfoliated graphene exhibits good electro-catalytic activity to the oxidation of Tyr, since it enhances the peak current and lowers the peak potential. In order to demonstrate the possible application of this electrochemical sensor, we used the standard addition method for the determination of Tyr in spiked samples, demonstrating excellent results.

## 2. Materials and Methods

### 2.1. Instruments

In order to investigate the morphological characteristics of graphene sample, a SEM/TEM Hitachi HD2700 instrument (Hitachi, Japan) equipped with a cold field emission gun (CSEG), operated at 200 kV and coupled with a double cut windowless 100 EDX detector acted by AZtec Software (Oxford Instruments) was used.

The X-ray powder diffraction (XRD) pattern was obtained with a Bruker D8 Advance Diffractometer using CuK_α1_ radiation (λ = 1.5406 Å). The background corrected pattern was used for the calculation of graphene structural parameters (inter-layer distance—d; crystallite size—D; number of layers—n)

The FTIR spectrum of graphene sample (mixed with KBr) was recorded with a JASCO 6100 FTIR spectrometer (4000–400 cm^−^^1^ spectral domain; 4 cm^−^^1^ resolution).

The UV-Vis spectrum of graphene powder was obtained with a SPECORD 250 PLUS instrument (Analytikjena). The sample was dispersed by ultrasound in double-distilled water for 3 min, to form a homogeneous suspension. Next, the spectrum was recorded within 200–800 nm range.

For electrochemical measurements (Cyclic Voltammetry—CV, Linear Sweep Voltammetry—LSV and Electrochemical Impedance Spectroscopy—EIS) a three-electrode cell coupled with a Potentiostat/Galvanostat Instrument (PGSTAT-302N, Metrohm-Autolab B.V., The Netherlands) was used. The CVs and LSVs measurements were generally run from 0.0 to +1 V vs. Ag/AgCl, at 10 mV/s scanning rate. EIS measurements were recorded between 0.1 and 10^5^ Hz, in solution containing 10^−^^3^ M potassium ferrocyanide and 0.2 M KCl.

For standard addition method, the following procedure was applied. A stock solution of 5 × 10^−^^4^ M Tyr was prepared in pH 7 PBS. Next, 0.1 mL of the stock was mixed with 4.9 mL pH 7 PBS which gave a final concentration of 1 × 10^−^^5^ M Tyr. This was denoted the unknown concentration (Cx). Next, three volumes (e.g., 100, 200, and 300 µL) of the stock solution (5 × 10^−^^4^ M) were added to three beakers, each also containing the unknown concentration Cx (the final volume in all beakers was 5 mL).

### 2.2. Chemicals

All chemicals, including: ammonia (Reactivul Bucuresti, Romania), ammonium thiocyanate (Sigma-Aldrich, Sternheim, Germany), potassium chloride (Reactivul Bucuresti, Romania), potassium ferrocyanide (Sigma-Aldrich, Sternheim, Germany), L-Tyrosine (Sigma-Aldrich, Sternheim, Germany) and Dimethylformamide (DMF) (JTBaker, HPLC grade, Sternheim, Germany) were used without further purification. The electrolyte solutions were prepared with double-distilled water produced with Fistreem Cyclon equipment.

### 2.3. Electrochemical Exfoliation of Graphite Rods for Graphene Synthesis (EGr)

Two electrochemical cells each containing two graphite rods and filled with 100 mL solution of electrolyte (0.1 M ammonia + 0.1 M ammonium thiocyanate) were connected to the exfoliation system (home-made system). A constant voltage of 11 V was applied between the graphite rods (anode and cathode) for about 4 h. In order to avoid the over-heating of the electrolyte, short pulses of current were applied for 0.8 s, followed by short pauses (0.2 s). The black powder deposited after exfoliation at the bottom of each cell was collected, washed with distilled water (10 L) and finally dispersed by ultrasound for 30 min in 125 mL water. Next, the black suspension was filtered on white-ribbon paper to remove the large particles and finally dried by lyophilization. The obtained sample was following denoted EGr.

### 2.4. Preparation of Graphene-Modified Electrode (EGr/GC)

In 2 mL solution of N,N-dimethylformamide (DMF) were dispersed by sonication 2 mg of graphene powder (3 min with a finger device; SONICS Vibra-Cell). Next, a volume of 10 µL from the dispersion was deposited by drop-casting onto the clean surface of a glassy carbon (GC) electrode and dried at room temperature for 24 h. After that, the modified electrode (EGr/GC) was employed for the electrochemical detection of Tyr.

## 3. Results and Discussion

### 3.1. Morphological and Structural Characterization of EGr Sample

**Figure 1:**
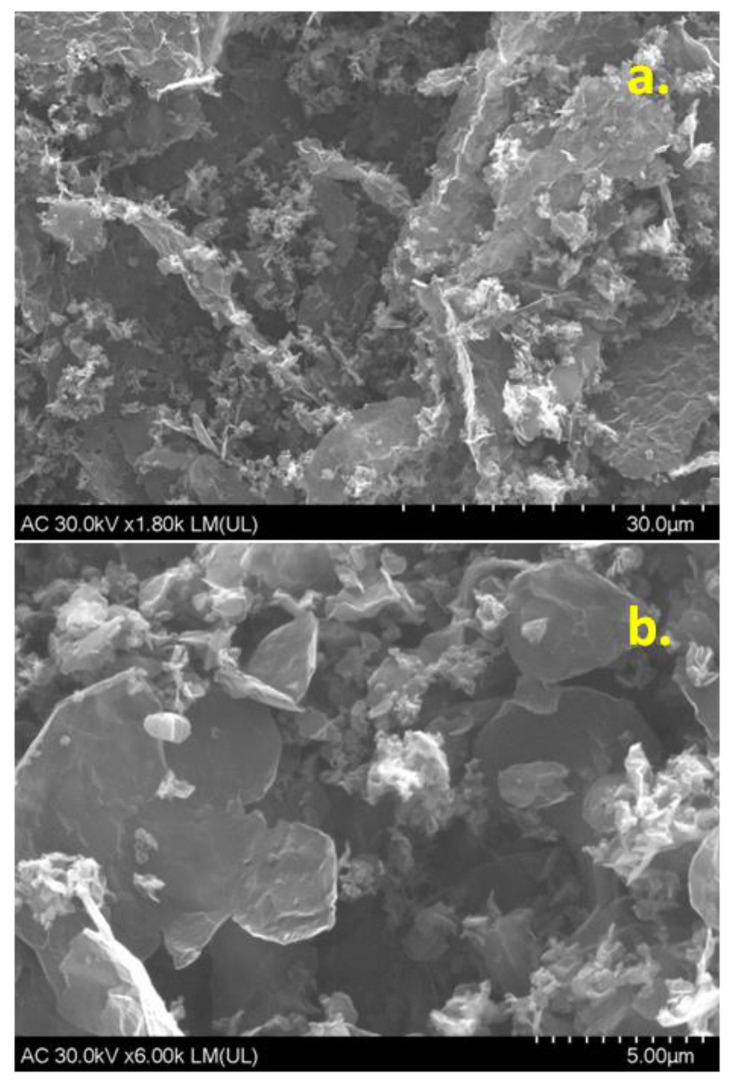
Representative SEM micrographs of EGr sample; scale bar: 30 µm (**a**); 5 µm (**b**).

The morphology of graphene powder deposited onto a solid substrate was investigated by scanning electron microscopy technique. Two representative micrographs are presented in Figure 1a,b, showing the porous appearance of the material. The high porosity brings a major advantage, such as the increase of the active area of the modified electrode and consequently of the electrochemical current. The micrograph with the higher resolution (Figure 1b) shows large and smooth areas corresponding to the basal plane of graphene as well as white lines (edges) where graphene layers fold-up.

**Figure 2:**
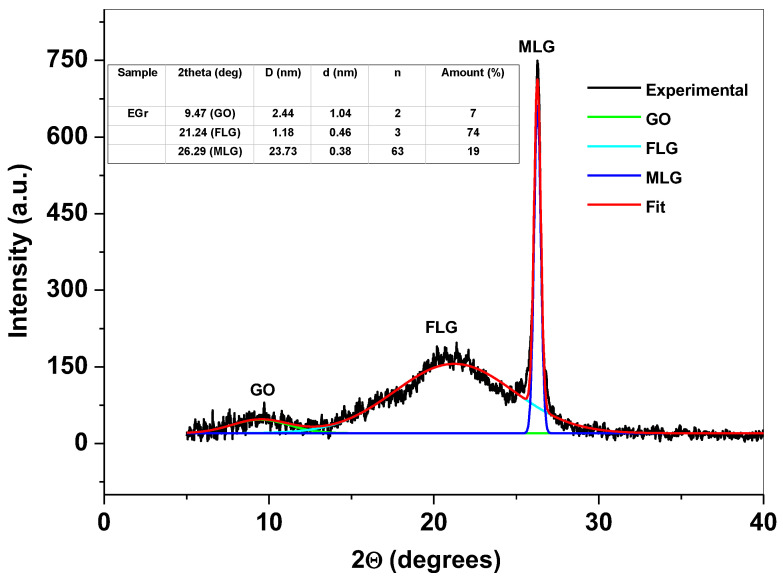
The XRD pattern of EGr sample, showing the presence of GO, FLG, and MLG.

The structure of graphene sample was next investigated by X-ray powder diffraction technique. In Figure 2 is presented the recorded pattern of the sample, evidencing three main peaks: the first one (at 9.47°) is small and broad and corresponds to the reflections of graphene oxide (GO) layers; the second one (at 21.24°) is also broad but of higher intensity and corresponds to the reflections of few-layer graphene (FLG); the third peak (at 26.29°) is due to the presence of multi-layer graphene (MLG) within the synthesized material. Using the experimental data, we determined the average number of graphene layers within the graphene crystallites (n), the inter-layer spacing (d), the mean crystallite size of graphene (D) and the amount of GO, FLG and MLG (expressed as %) present within the sample [30,31,32]. The results are presented in the inset of Figure 2.

**Figure 3:**
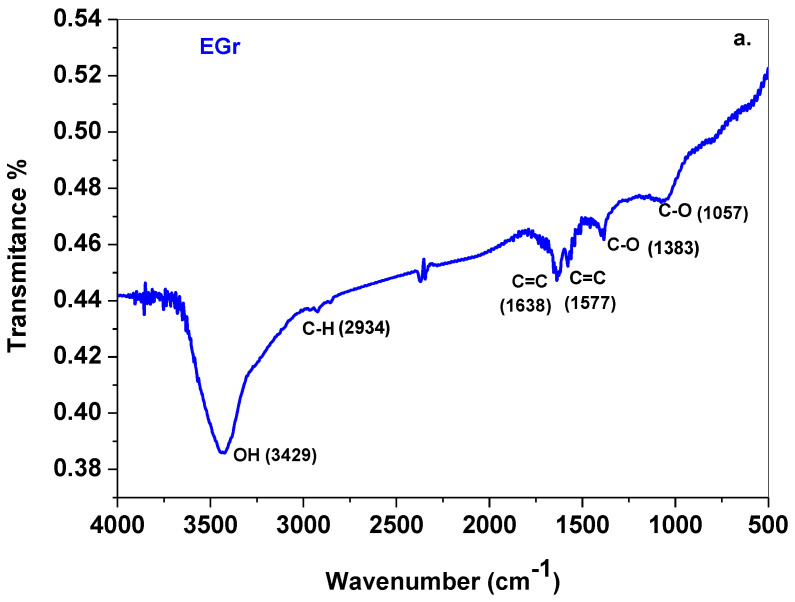

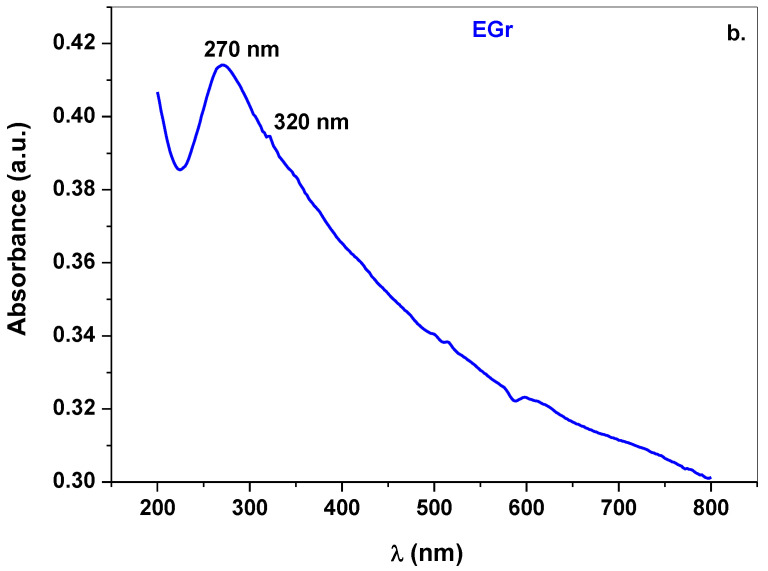
The FTIR spectrum of EGr sample (**a**); the UV-Vis spectrum of the EGr sample (**b**).

Next, the vibration characteristics of the synthesized sample were evidenced by FTIR spectroscopy. The corresponding spectrum is illustrated in Figure 3a. The presence of some oxygen-containing functional groups can be observed, in good agreement with the literature [33]. The main adsorption band, at 3429 cm^−^^1^ is assigned to the O-H stretching vibrations from the adsorbed water molecules, while the two adsorption peaks at 1383 and 1057 cm^−^^1^ are assigned to the C-O stretching vibrations. Their weak intensity is in excellent agreement with the XRD pattern of the sample where a small GO peak is evidenced. The peaks at 1577 cm^−^^1^ and 1638 cm^−^^1^ represents the vibration modes of sp^2^-hybridized C=C. Complementary to this, the UV-Vis spectrum of the sample (Figure 3b) reveals a broad peak at 270 nm, due to π-π* transitions of electrons within C=C bonds. The lack of a well-defined peak at 320 nm (due to n-π* transitions of electrons within C-O bonds) indicates that the sp^2^ configuration of carbon atoms is predominant within the synthesized material.

### 3.2. Electrochemical Studies

In order to fully characterize the bare and graphene-modified electrodes, the EIS spectra were recorded and the experimental data were represented as Nyquist plots (Figure S1). The charge-transfer resistance (R_ct_) values were determined after fitting the Nyquist plots with the appropriate equivalent electrical circuit. The first circuit characterizes the bare GC electrode and contains the following elements: the solution resistance (R_s_) which depends on the concentration of supporting electrolyte, the Warburg impedance (Z_W_) that appears due to the diffusion of ions within the double-layer at low frequency, the charge-transfer resistance (R_ct_) that reflects the easiness of electron transfer across electrode-solution interface and a constant phase element (CPE) that replaces the double-layer capacitance (C_dl_). For graphene-modified electrode, due to the high porosity of graphene layer the circuit additionally contains a transmission line (T) [34]. After fitting the data, the R_ct_ values were determined to be: 75.5 kΩ for bare GC and 1.16 kΩ for EGr/GC, indicating that the graphene layer highly favors the transfer of electrons when deposited on top of a conductive substrate.

**Figure 4:**
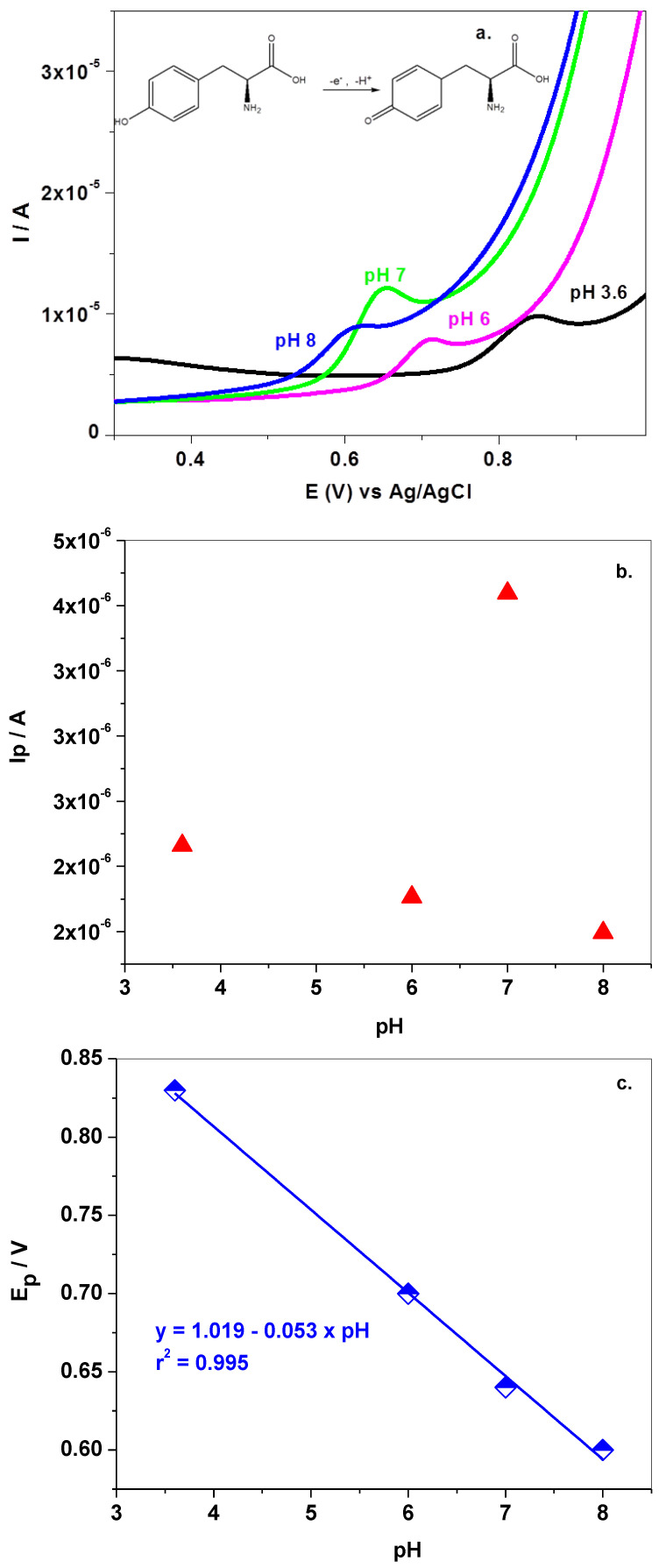
LSVs recorded with graphene-modified electrode in solutions of various pH (3.6–8), each containing 10^−^^4^ M Tyr; 10 mV/s scanning rate (**a**); variation of peak current (I_p_) with the solution pH (**b**); variation of peak potential (E_p_) with the solution pH (**c**).

Next, the electrochemical oxidation of Tyr (10^−^^4^ M) was investigated in buffer solutions of various pH (3.6–8), using the LSV technique (Figure 4a–c). The signal correlates well with the pKa of this amino-acid (pK_a1_= 2.20 for α-carboxyl group; pK_a2_ = 9.11 for α-ammonium ion). In highly acidic solution (pH 3.6) a clear oxidation peak appears at around +0.85 V which shifts towards lower potentials, in neutral and basic solutions. The linear regression equation that describes the variation of peak potential, E_p_, versus pH is given by: y = 1.019 − 0.053 × pH. In this case, the slope value is 0.053 V/pH being close to that obtained from a Nernstian plot (0.059 V/pH) and indicating an equal number of protons and electrons involved in the electrochemical reaction. The maximum peak current, I_p_, was observed in pH 7 PBS, so all of the experiments were next performed at this pH (Figure 4b,c).

**Figure 5:**
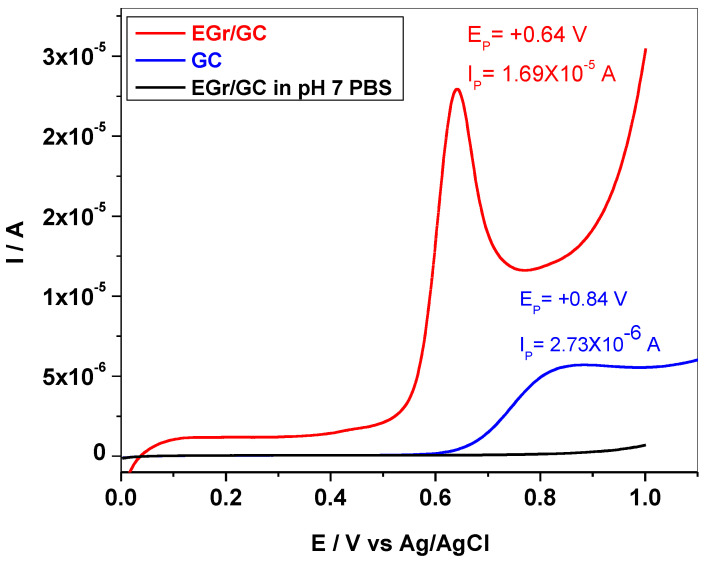
LSVs recorded with EGr/GC (red) and GC (blue) electrodes in pH 7 PBS containing 10^−^^3^ M Tyr; the black curve represents the background current for EGr/GC electrode (in pH 7 PBS); 10 mV/s scanning rate.

In order to evidence the electro-catalytic properties of the synthesized material towards L-tyrosine oxidation, the comparison between the graphene-modified electrode and the bare GC electrode is shown (Figure 5). The LSVs were recorded in pH 7 PBS containing 10^−^^3^ M Tyr, revealing marked differences between the two electrodes. Hence, in the case of bare GC, the oxidation peak is broad and of low intensity (2.73 × 10^−^^6^ A) and appears at higher potential (+0.84 V) in comparison with that of graphene-modified electrode (+0.64 V). The increased sensitivity of EGr/GC towards Tyr oxidation may be due to the enhanced electron transfer rate at the edges of graphene sheets. In addition, the π–π stacking interaction between the aromatic ring of Tyr and graphene may help with the orientation of OH group towards graphene, favoring its oxidation.

**Figure 6:**
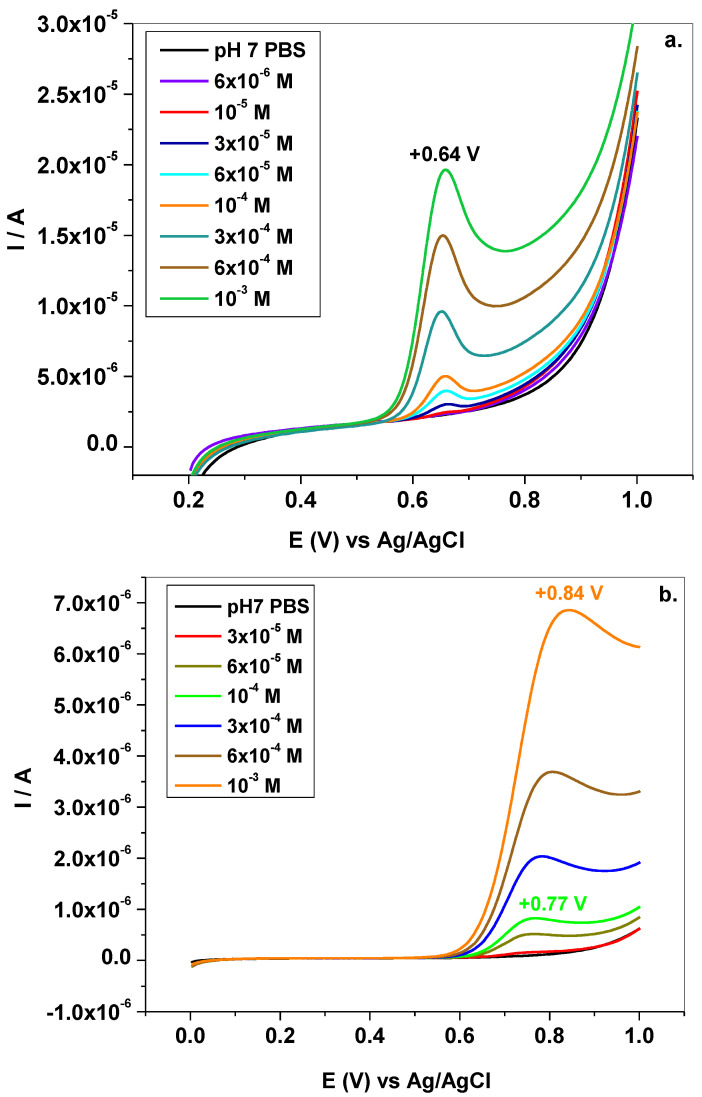
LSVs recorded with EGr/GC (**a**) and GC (**b**) electrodes in pH7 PBS containing various concentration of Tyr: 6 × 10^−^^6^–10^−^^3^ M range (**a**); 3 × 10^−^^5^–10^−^^3^ M range (**b**); 10 mV/s scanning rate.

Next, Tyr was quantitatively analyzed by LSV and the corresponding recordings at various concentrations are plotted in Figure 6a,b. There are significant differences between the two electrodes which are worth mentioning. In the case of EGr/GC electrode, the oxidation potential (+0.64 V) does not change with Tyr concentration, indicating a weak adsorption of the oxidation products on its surface. In contrast, for GC electrode, the peak potential is highly dependent on concentration, varying from +0.77 to +0.84 V. In addition, the lowest signal recorded with EGr/GC electrode was obtained in solution of 6 × 10^−^^6^ M Tyr, while with bare GC the lowest signal was obtained at a higher concentration, of 3 × 10^−^^5^ M Tyr. This indicates an increased sensitivity of the graphene-modified electrode, in comparison with bare GC.

**Figure 7:**
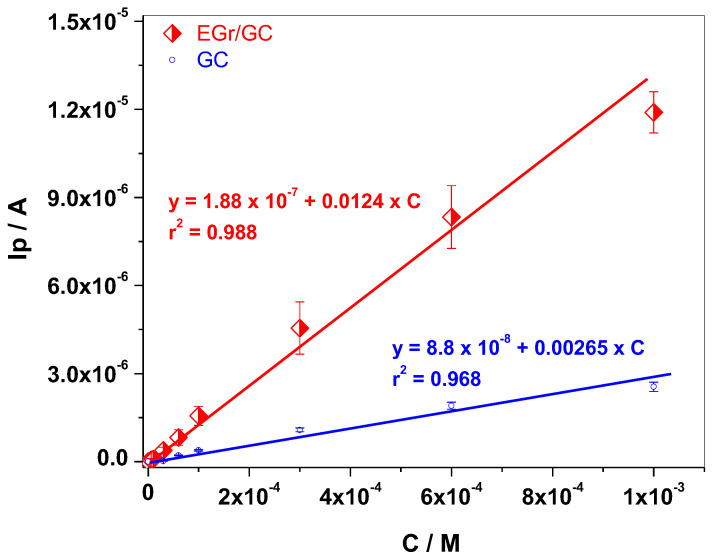
Calibration plot obtained with EGr/GC electrode, within 6 × 10^−^^6^–10^−^^3^ M range Tyr, in pH 7 PBS (red curve); Calibration plot obtained with GC electrode, within 3 × 10^−^^5^–10^−^^3^ M range Tyr, in pH 7 PBS (blue curve).

By representing the peak current (I_p_) versus Tyr concentration, the corresponding calibration plots were obtained and represented in Figure 7. The linear regression equation for EGr/GC electrode is: I_p_ = 1.88 × 10^−^^7^ + 0.0124 × C, R^2^ = 0.988, while for bare GC is I_p_ = 8.8 × 10^−^^8^ + 0.00265 × C, R^2^ = 0.968. As can be seen in this figure, the EGr/GC electrode has a considerably higher sensitivity (0.0124 A/M) and lower detection limit (1.81 × 10^−^^6^ M), in comparison with those of bare GC: 0.00264 A/M sensitivity and 9 × 10^−^^6^ M detection limit. The detection limit was obtained by dividing the limit of determination (LOD) by 3.3.

**Figure 8:**
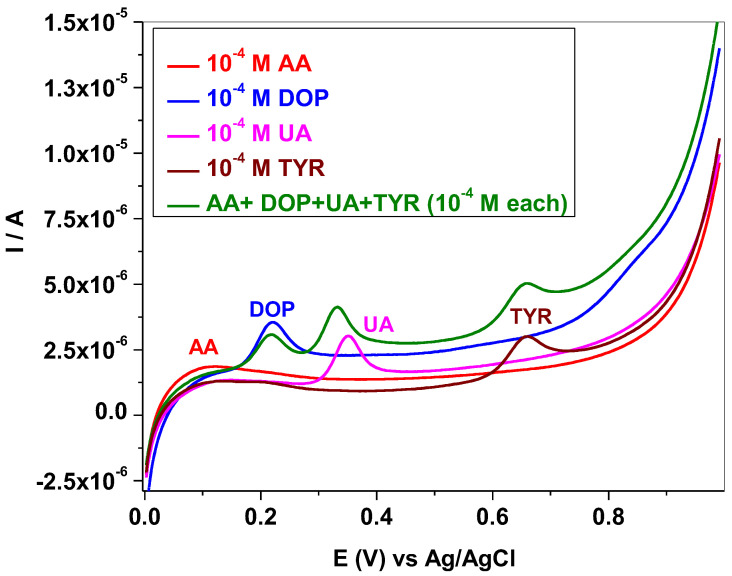
LSVs recorded with EGr/GC electrode in pH7 PBS containing single analyte: 10^−^^4^ M ascorbic acid (red curve); 10^−^^4^ M dopamine (blue curve); 10^−^^4^ M uric acid (pink curve); 10^−^^4^ M Tyr (brown curve); and a mixture of all (AA + DOP + UA + TYR), 10^−^^4^ M each (olive curve); 10 mV/s scanning rate.

The selectivity of EGr/GC electrode towards Tyr oxidation was evaluated in the presence of other biomolecules, such as ascorbic acid (AA), dopamine (DOP) and uric acid (UA). In Figure 8 are presented the LSV curves recorded for a single analyte and for their mixture (10^−^^4^ M each). In the case of a single analyte (ascorbic acid) the oxidation signal appears as a very broad peak, at low potential (+0.16 V; red curve). The dopamine peak is well defined and appears at higher potential (+0.22 V; blue curve) while uric acid appears at +0.35 V (pink curve). When Tyr is mixed with all of these analytes (10^−^^4^ M each), an increase of the capacitive current is observed but no changes are noticed for the peak potential (see the olive curve in comparison with the brown curve). For the Tyr peak intensity, a slight increase is observed, from 1.042 × 10^−^^6^ A (TYR- single analyte) to 1.129 × 10^−^^6^ A (mixture of TYR + AA + DOP + UA), indicating a weak influence of the interfering species.

**Figure 9:**
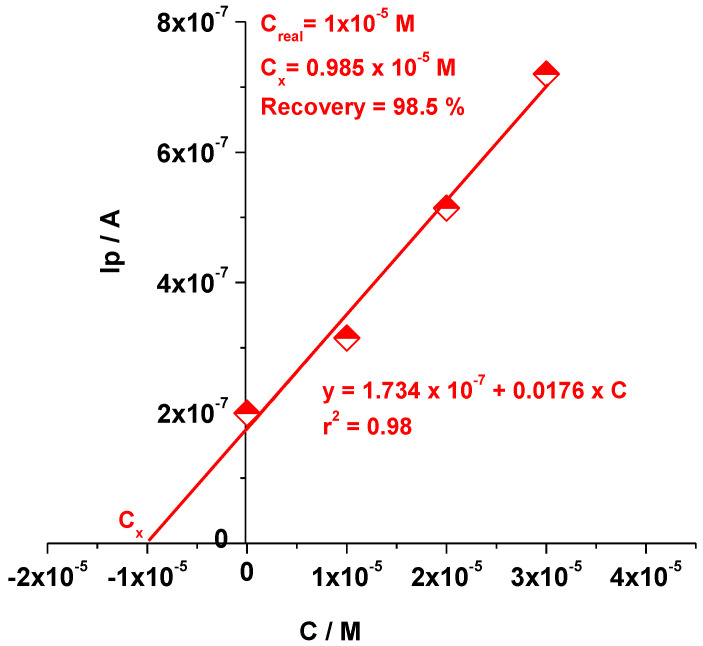
The standard addition plot obtained for Tyr detection.

The EGr/GC electrode was also tested for Tyr detection using the standard addition method (Figure 9). The peak current obtained from the LSV recordings was plotted versus Tyr concentration, as can be seen in Figure 9, allowing us to determine Cx. In this case Cx was found to be 0.985 × 10^−5^ M, giving an excellent recovery (98.5 %). In order to confirm the results five standard addition measurements were performed and the recovery range was between 98.5% and 103.1%, proving the efficiency of the EGr/GC electrode.

Several advantages of the employed electrode can be mentioned such as the simple approach used for manufacturing the graphene-modified electrode, the very wide linear range of L-Tyrosine with good sensitivity and detection limit, and the excellent recovery results obtained with the standard addition method in spiked samples (Table 1).

In addition, it is important to mention that the graphene sample employed as electro-catalyst has an excellent time stability. If stored in a closed recipient it can be used in electrochemistry for over a year. As concerned the modified electrode, it shows an excellent working sensitivity. More than 20 measurements were recorded with the same electrode and the signal was >95% of its original value. Between the measurements, the electrode was kept in distilled water for 15 min to desorb the surface contaminants. The longtime stability was also investigated over a 10 days period, indicating that the analytical performance of the modified electrode has not significantly changed (signal > 95%), Figure S2.

## 4. Conclusions

A graphene sample was prepared by electrochemical exfoliation of graphite rods in solution containing 0.1 M ammonia + 0.1 M ammonium thiocyanate. The porous morphology of the sample was revealed by SEM technique while the structural parameters were determined from the XRD pattern. Hence, the average number (n) of layers within the graphene crystallites, the inter-layer spacing (d), the mean crystallite size (D) of graphene and the amount of GO, FLG, and MLG (expressed as %) were determined. In addition, FTIR and UV-Vis complementarily characterized the sample indicating that the sp^2^ configuration of carbon atoms is predominant within the synthesized material. The graphene sample was used for glassy carbon surface modification (EGr/GC) and next tested towards Tyr detection. By representing the peak current (I_p_) versus Tyr concentration, the corresponding calibration plots were obtained. The linear regression equation for EGr/GC electrode was: I_p_ = 1.88 × 10^−7^ + 0.0124 × C, R^2^ = 0.988, while for bare GC was I_p_ = 8.8 × 10^−8^ + 0.00265 × C, R^2^ = 0.968. As expected, the EGr/GC electrode had higher sensitivity (0.0124 A/M) and lower detection limit (1.81 × 10^−6^ M), in comparison with those of bare GC: 0.00264 A/M sensitivity and 9 × 10^−6^ M detection limit.

## Figures and Tables

**Table 1:** Comparison of EGr/GC with other modified electrodes employed for the determination of Tyr.

Sensing Material/Electrode	Linear Range (M)	DL (M)	Sensitivity A/M	Recovery %	Ref.
Nano-Au-/CA/GCE	10^−7^ M−3 × 10^−4^	4 × 10^−8^	-	99.9–102.4	[10]
GR/Au NPs/GCE	0.1–100 × 10^−6^	47 × 10^−9^	0.918	-	[29]
GR/ZnO/SPE	10^−6^–8 × 10^−4^	3.4 × 10^−7^	0.016	97.5–103	[35]
ERGO/GCE	0.5–80 × 10^−6^	0.2 × −10^−6^	0.026	-	[36]
PPy/FeCN-SPCE	0.5–27 × 10^−6^	8.20 × 10^−8^	1.46	99.92–103.97	[37]
PPy/NP-SPCE		4.30 × 10^−7^	0.278		
PPy/SDS-SPCE		3.51 × 10^−7^	0.341		
Al-CuSe-NPs/SPCE	0.15–10 × 10^−6^	0.04 × 10^−6^	0.053	97.6–101	[38]
EGr/GC	6 × 10^−6^–10^−3^	1.81 × 10^−6^	0.0124	98.5–103.1	This work

## Data Availability

Data will be provided upon reasonable request to the corresponding author.

## Supplementary Materials

The following supporting information can be downloaded at: https://www.mdpi.com/article/10.3390/s22103606/s1, Figure S1. Equivalent electrical circuits employed to fit the experimental results obtained with bare GC and graphene-modified electrode (a); Nyquist plots obtained for bare GC (blue) and EGr/GC (red) electrodes in solution containing 1.0 mM K4[Fe(CN)6] + 0.2 M KCl; applied potential: +0.25 V (b).; Figure S2. The longtime stability investigated over a 10 days period with EGr/GC electrode (5 × 10^−4^ M Tyr in pH 7 PBS).
