# Supplementary material for: Electrochemical L-Tyrosine Sensor Based on a Glassy Carbon Electrode Modified with Exfoliated Graphene
Source: Sensors (Basel). 2022 May 10;22(10):3606. doi: 10.3390/s22103606 (PMC9143931; doi:10.3390/s22103606)
Supplement: Supplementary file 1 [file sensors-22-03606-s001.zip › sensors-1709843-supplementary.pdf]

sensors

MDPI

Article

Electrochemical L-tyrosine Sensor Based on a Glassy Carbon Electrode Modified with Exfoliated Graphene

Codruţa Varodi, Florina Pogăcean *, Maria Coroş, Alexandra Ciorîță and Stela Pruneanu *

National Institute for Research and Development of Isotopic and Molecular Technologies, Donat Street, No. 67-103, 400293 Cluj-Napoca, Romania; codruta.varodi@itim-cj.ro (C.V.); maria.coros@itim-cj.ro (M.C.); alexandra.ciorita@itim-cj.ro (A.C.)

* Correspondence: florina.pogacean@itim-cj.ro (F.P.); stela.pruneanu@itim-cj.ro (S.P.)

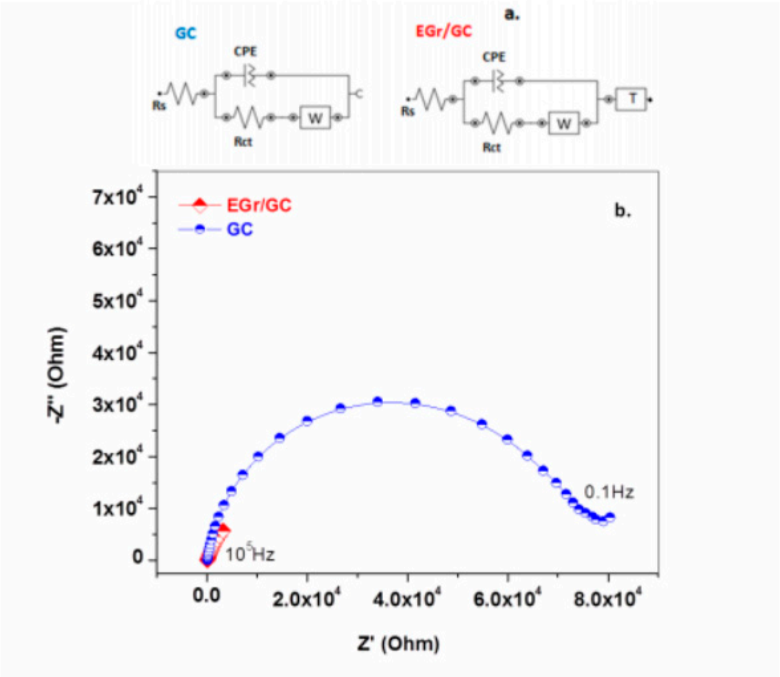

Figure S1. Equivalent electrical circuits employed to fit the experimental results obtained with bare GC and graphene-modified electrode (**a**); Nyquist plots obtained for bare GC (blue) and EGr/GC (red) electrodes in solution containing 1.0 mM K4[Fe(CN)6] + 0.2 M KCl; applied potential: +0.25 V (**b**).

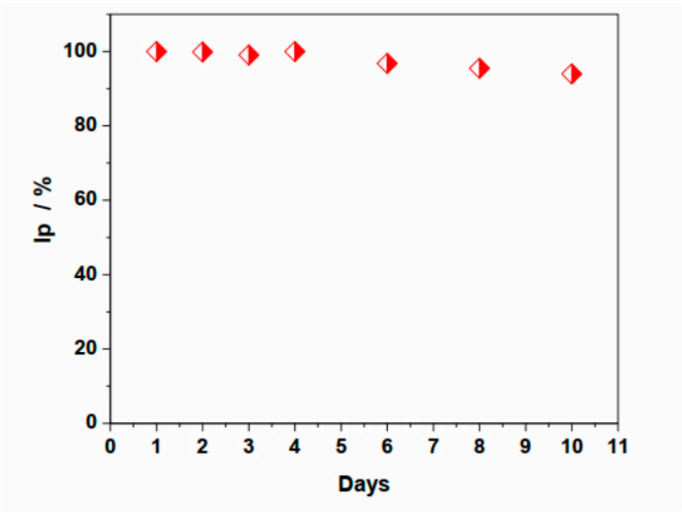

Figure S2. The longtime stability investigated over a 10 days period with EGr/GC electrode (5×10^{-4} M Tyr in pH 7 PBS).
